# Impact of Gastrointestinal *Bacillus anthracis* Infection on Hepatic B Cells

**DOI:** 10.3390/toxins7093805

**Published:** 2015-09-22

**Authors:** Natacha Colliou, Bikash Sahay, Mojgan Zadeh, Jennifer L. Owen, Mansour Mohamadzadeh

**Affiliations:** 1 Department of Infectious Diseases and Pathology, College of Veterinary Medicine, University of Florida, Gainesville, FL 32608, USA; E-Mails: collioun@ufl.edu (N.C.); sahayb@ufl.edu (B.S.); zadehm@ufl.edu (M.Z.); 2 Division of Gastroenterology, Hepatology, and Nutrition, College of Medicine, University of Florida, Gainesville, FL 32608, USA; 3 Department of Physiological Sciences, College of Veterinary Medicine, University of Florida, Gainesville, FL 32610, USA; E-Mail: jlowen@ufl.edu

**Keywords:** B cells, gastrointestinal anthrax, innate lymphoid cells, liver

## Abstract

Ingestion of *Bacillus anthracis* results in rapid gastrointestinal (GI) infection, known as GI anthrax. We previously showed that during GI anthrax, there is swift deterioration of intestinal barrier function leading to translocation of gut-associated bacteria into systemic circulation. Additionally, we described dysfunction in colonic B cells. In concordance with our previous studies, here, we report early migration of the Sterne strain of *B. anthracis* along with other gut-resident bacteria into the infected murine liver. Additionally, despite a global decrease in the B cell population, we observed an increase in both B-1a and marginal zone (MZ)-like B cells. Both of these cell types are capable of producing immunoglobulins against common pathogens and commensals, which act as a general antibody barrier before an antigen-specific antibody response. Accumulation of these cells in the liver was associated with an increase in chemokine expression. These data suggest that the presence of Sterne and other commensals in the liver trigger migration of MZ-like B cells from the spleen to the liver to neutralize systemic spread. Further research is required to evaluate the possible cause of their failure to clear the infection within the liver, including the potential role of dysfunctional mitogen-activated protein kinase (MAPK) signaling.

## 1. Introduction

*Bacillus anthracis*, a Gram-positive and spore-forming bacterium, is the causative agent of anthrax, an infectious disease affecting all warm-blooded animals, including humans [1]. The bacterium hosts a single chromosome and two large extra-chromosomal plasmids, pXO1 (182kb) and pXO2 (96kb), which are essential for full virulence. The genes on the pXO1 plasmid encode components of the exotoxin complex, responsible for clinical manifestations of the disease, whereas the pXO2 plasmid encodes enzymes for the synthesis of a unique anti-phagocytic capsule made up of poly-d-glutamic acid [2,3,4,5]. The bacterium utilizes one of three routes to enter the body, leading to categorization of the disease into three distinct types: cutaneous, gastrointestinal, and inhalational anthrax [6]. Most previous studies have focused on inhalational and cutaneous anthrax [7,8]. Due to the presence of the resident gut microbiota and unique cells in the GI tract, the disease manifests differently with GI anthrax. Previously, we reported that *B. anthracis* Sterne strain-gavaged A/J mice exhibited lethal infection with a systemic spread of pathogens, including some gut-associated bacteria due to a compromised intestinal mucosal barrier [9]. Additionally, we described impaired antibody production by innate B cell populations in the gut [10].

Herein, we describe how oral infection with *B. anthracis* Sterne leads to the migration of gut-associated bacteria into the liver, with subsequent migration to other organs, suggesting a hematogenous route of dissemination. Additionally, the impact of infection on B cells within the liver is very different from that seen in the gut, published earlier by our laboratory [10]. Here, we report a general depletion of B cells with hepatic infection; however, an increase in B-1a and marginal zone-like B cells within the liver parenchyma was observed at later stages of the disease. This increase in B cells correlates with the increased expression of B cell chemoattractants in the liver. Moreover, type 2 innate lymphoid cells (ILC2s) increased with hepatic infection; these cells were shown to be depleted in the gut in previous studies of GI anthrax [10].

## 2. Results and Discussion

### 2.1. B. anthracis Sterne Infection Allows Dissemination of Gut-Associated Microbes within the Liver

**Figure 1:**
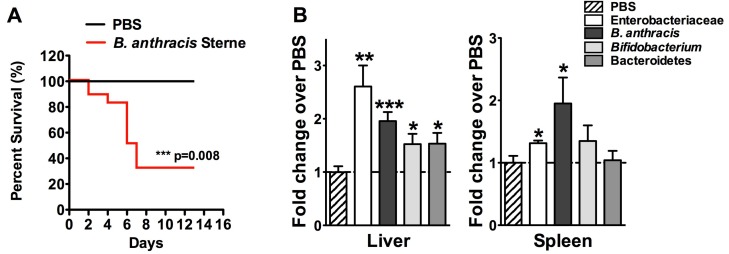
Lethal oral infection with *Bacillus anthracis* Sterne causes a polymicrobial infection systemically. (**A**) A/J mice (*n* = 13) were orally gavaged with 10^9^ spores of the *B. anthracis* Sterne and survival data were graphed; (**B**) Livers and spleens of infected mice (*n* = 13) were excised 5 days post-infection and DNA was isolated. The relative presence of bacterial DNA was measured using specific primers described in the Experimental Section. * *p* < 0.05, ** *p* < 0.01, *** *p* < 0.001 compared to PBS.

Due to deficiency of the complement component, C5a, the A/J strain of mice is susceptible to infection with *B. anthracis* Sterne [11], which lacks the capsule that protects the bacteria against phagocytosis [12]. Mice were gavaged with *B. anthracis* Sterne spores (10^9^ CFU/mouse) to evaluate the disease process. Two days post-infection, A/J mice began to exhibit lethargy and signs of dyspnea. At day 14, 9/13 (69.2%) of infected mice succumbed to infection (*p* = 0.008) (Figure S1). We had reported earlier that Sterne infection in mice leads to a decline in intestinal barrier function, resulting in the systemic spread of the Sterne bacterium and gut-associated bacteria [9]; thus, we sought to evaluate bacterial spread within the liver. We quantified the presence of *B. anthracis*, Enterobacteriaceae, *Bifidobacterium*, and Bacteroidetes by quantitative real-time PCR (qRT-PCR) using specific primer sets in the livers and the spleens of mice five days post-infection (Figure 1B). In the livers of the orally infected mice, those bacteria were present in significant number at day five post-infection compared to the control mice; however, only the Sterne strain (*B. anthracis*) and Enterobacteriaceae were increased in number in the spleen at that time point. The increased presence of bacterial species in the liver suggests a route of dissemination via the portal vein. The liver is a unique organ in that it receives blood from two sources, (1) from the general circulation of oxygenated blood via the hepatic artery and (2) from the portal vein that delivers absorbed nutrients for further processing via hepatocellular enzymes [13]. The portal vein not only brings nutrients from the intestines, it also infuses the liver with metabolites generated at intestinal locations, bacterial products like lipopolysaccharide (LPS), and sometimes, intact bacteria [14]. The survival of bacteria in the liver is limited due to the presence of capable immune cells, such as Kupffer cells, hepatocytes, and T and B lymphocytes [13,15]; however, during a breach in the intestinal epithelial barrier, a significant number of gut bacteria has been observed and can contribute to liver pathology, including cirrhosis [16,17]. Similar to previously described intestinal pathology, GI Sterne infection resulted in a compromised intestinal barrier, allowing the bacteria normally contained within the intestinal lumen to enter the systemic circulation via the portal vein.

### 2.2. Characterization of B Cell Subpopulations in the Liver of A/J Mice

B cells make up approximately 10% of human intrahepatic lymphocytes; however, in the murine liver, approximately 20%–50% of the intrahepatic lymphocytes are B cells [18,19]. There are different types of B cells present in the murine liver, and to characterize the different phenotypes, we employed an extensive flow cytometry strategy to identify these B cell subsets [20]. The strategy we utilized is based on a combination of antibodies that allowed us to distinguish all B cell subsets present in the spleen and liver. Gated on CD19^+^B220^+^ B cells, we identified different B cell subsets using a combination of antibodies (CD19, B220, CD23, CD21, IgM, and IgD). Different expression levels of these markers allowed us to discriminate Transitional (T1 and T2) B cell subsets; e.g., T1 (B220^+^CD19^+^IgD^low^IgM^low^CD23^−^CD21/35^−^), and T2 (CD19^+^IgD^high^CD23^high^IgM^high^CD21/35^low^). The originally described “T2” subset contained MZ B cell precursors (MZP) (B220^+^CD19^+^IgD^high^CD21/35^high^CD23^+^IgM^high^) that differ due to the presence of CD21/35 surface markers. T2-MZP cells also expressed CD23, which permits differentiation from MZ B cells (B220^+^CD19^+^IgD^low^CD21/35^high^CD23^−^IgM^high^). Long-lived recirculating follicular B cells (FO, also called B-2 cells) can also be identified (B220^+^CD19^+^IgM^low^CD23^+^CD21/35^low^IgD^high^) (Figure S1). The characterization of B-1 cells was based on the use of CD19^+^IgM^high^CD23^low(−)^CD21^−^IgD^low^CD11b^+^ and CD5^+/−^^(B−1a/B−1b)^ staining.

**Figure 2:**
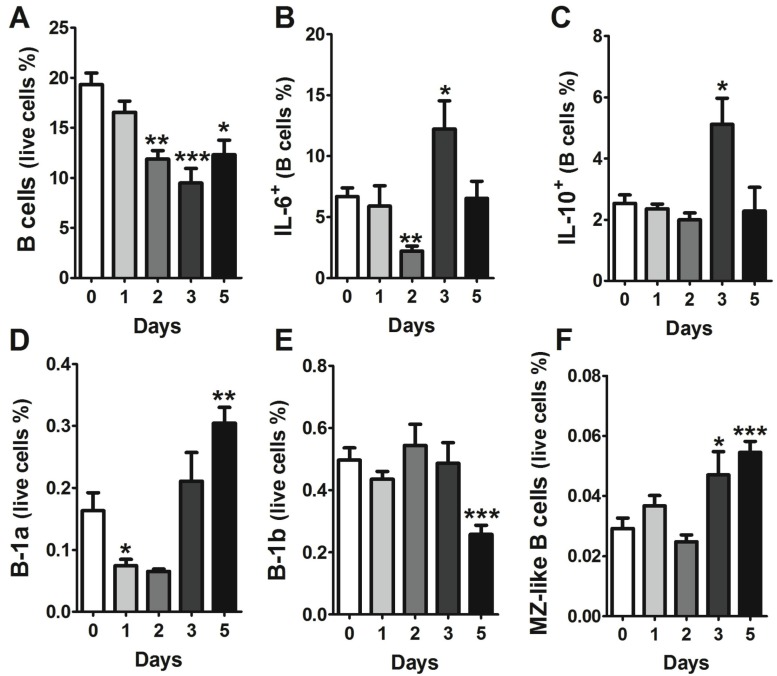
Sterne infection causes changes in the B cell population in the liver. A/J mice (*n* = 43) were infected with 10^9^ spores of *B. anthracis* Sterne. Liver leukocytes were isolated from mice (day 1, *n* = 10; day 2, *n* = 10, day 3, *n* = 10; day 5 *n* = 13) at each indicated time point using Percoll gradients, as described in the Experimental Section. Isolated leukocytes were stained with different sets of antibodies, as depicted in the Figure S1 to define: (**A**) total B cells; (**B**) IL-6 producing; (**C**) IL-10 producing B cells; (**D**) B-1a cells; (**E**) B-1b cells; and (**F**) marginal zone-like (MZ-like) B cells. Data are shown as mean ± SEM. * *p* < 0.05, ** <0.01, *** *p* < 0.001 compared with PBS-treated or day 0 mice.

In our previous study, we showed that GI anthrax leads to a dysfunction in B-1 cells, which are a dominant B-cell population in the gut [10]; the significant presence of the Sterne strain bacteria in the livers of infected mice prompted us to further investigate B cell phenotypes in the livers of A/J mice gavaged with Sterne spores. B cells represented approximately 20% of the total leukocyte population in healthy murine livers; this percentage gradually decreased over time during oral infection with *B. anthracis* Sterne. However, at day five post-infection, a slight increase in the B cell population was observed compared to day three post-infection; nonetheless, this was significantly lower compared to the uninfected control liver samples (Figure S2). The mice that survived the infection and survived up to 14 days post-infection, recovered from the loss in the B cell population (18.45 ± 6.7) (data not shown). These changes in the B cell population were only observed in the innate B cell subpopulations; the major B cell populations (Follicular and T2 B cells) remained unaffected (Figure S2). Toxins secreted by the Sterne strain can effectively inhibit the transcription of cytokines (e.g., IL-6 and IL-10); however, these cytokines were significantly enhanced at day three post-infection (Figure 2B,C), which may suggest migration of unaffected healthy B cells into the liver from other organs that did not contain bacteria. To evaluate which types of B cells could migrate into the liver parenchyma, we performed careful analyses of different B cell populations in the liver and spleen, as described above. Global analyses of B cells revealed an initial decrease in B-1a cells, which later increased in their percentage within the liver parenchyma (Figure 2D). Conversely, B-1b cells maintained their presence during the initial phase of disease (up to three days post-infection); however, this population exhibited a sharp decline at five days post-infection (Figure 2E). Surprisingly, we were able to detect B cells that possessed, albeit at a low rate, a marginal zone (MZ)-like B cell phenotype (B220^+^CD19^+^IgD^low^CD21/35^high^CD23^−^IgM^high^) in the liver three days post-infection, and more significantly, five days after infection (0.054 ± 0.01 at day five *versus* 0.029 ± 0.01 for PBS mice, *p* < 0.001) (Figure 2F). So, similar to B-1a cells, MZ-like B cells exhibited an increased presence at days three and five post-infection (Figure 2). Functionally, B-1a and MZ-like B cells are similar, as they secrete non-specific immunoglobulins (IgM) to neutralize common pathogens and also produce IL-10, a key regulatory cytokine that plays a crucial role in downmodulating immune responses [21,22]. Migration of these unique cells is dependent upon the chemokine gradient and expression of specific chemokine receptors on these cells.

### 2.3. Evaluation of Chemokine and Chemokine Receptors in the Liver during B. anthracis Sterne Infection

**Figure 3:**
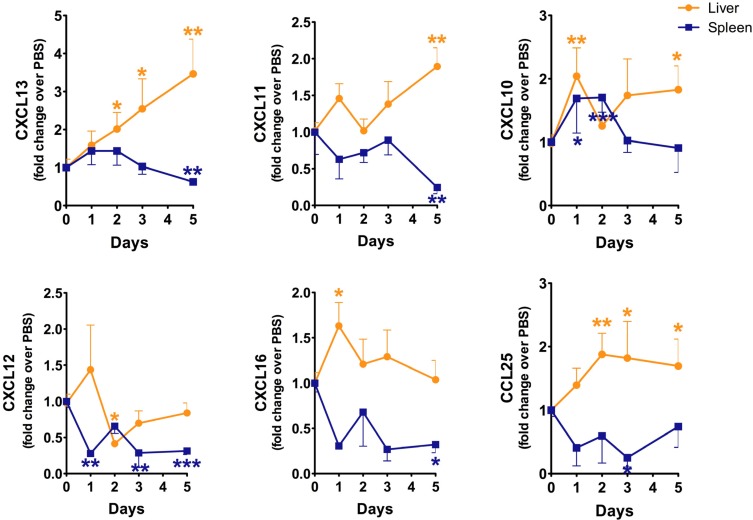
Sterne infection leads to changes in chemokine production in the liver and spleen. A/J mice (*n* = 43) were infected with 10^9^ spores of *B. anthracis* Sterne. Total RNA was isolated from mice (day 1, *n* = 10; day 2, *n* = 10, day 3, *n* = 10; day 5 *n* = 13) at each indicated time point, and transcripts of CXCL10, CXCL11, CXCL12, CXCL13, CXCL16, and CCL25 were evaluated by qRT-PCR, as mentioned in the Experimental Section using gene specific primers. Data are shown as mean ± SEM. * *p* < 0.05, ** <0.01, *** *p* < 0.001 compared with PBS-treated or day 0 mice.

The chemokines CXCL10; -11; -12; -13; and -16 have been implicated in either B cell trafficking or the infiltration of other immune cells into the liver [23,24,25,26,27]. To evaluate whether expression of these chemokines is altered with Sterne infection; we analyzed transcripts by qRT-PCR; which revealed differential expression of CXCL10; -11; -12; -13; and -16 in the liver and in the spleen during Sterne infection (Figure 3). CXCL10 and -11 (ligands for the CXCR3 receptor) were moderately expressed in the liver; however; their expression was downregulated in the infected spleen. The expression of these two chemokines is dependent upon interferon signaling; they then recruit inflammatory leukocytes to the inflamed loci [28]. It is conceivable that inflammation would be increased with polymicrobial infiltrates; as seen in the liver during Sterne infection. CXCL12 binds to its receptor; CXCR4; and is secreted in response to Toll-like receptor (TLR) and cytokine signaling; to recruit inflammatory leukocytes [29,30]; this chemokine was inhibited during Sterne infection. The classical chemokine for B cell recruitment; CXCL13; was significantly increased during Sterne infection in the liver; however; its expression in the spleen was moderately decreased at day five post-Sterne infection (Figure 3). Perturbation in chemokine expression in the liver and the spleen may form a temporary chemokine gradient for the migration of cells from a distant location; including from the spleen to the liver. Furthermore; the downregulation of homeostatic lymphoid chemokines in the spleen; such as CXCL13; could lead to disruption of the splenic architecture; resulting in the traffic of MZ-like B cells into the liver.

### 2.4. Sterne Infection Increases the Accumulation of Type 2 Innate Lymphoid Cells

**Figure 4:**
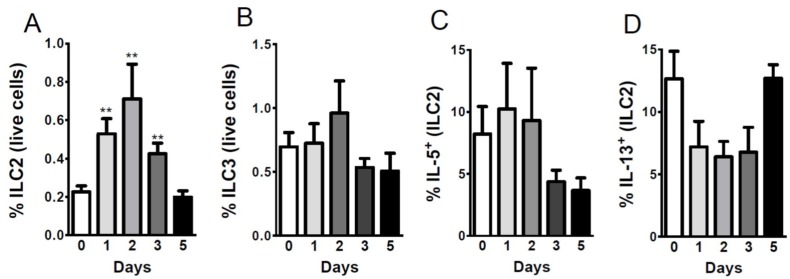
Sterne infection changes the percentage of ILC2s in infected livers. A/J mice (*n* = 43) were infected with 10^9^ spores of *B. anthracis* Sterne. Liver leukocytes were isolated from mice (day 1, *n* = 10; day 2, *n* = 10, day 3, *n* = 10; day 5 *n* = 13) at each indicated time point using Percoll gradients, as described in the Experimental Section. Isolated leukocytes were stained with different sets of antibodies, as depicted in the Figure S1 to define: (**A**) ILC2s; (**B**) ILC3s; (**C**) IL-5 producing; and (**D**) IL-13 producing ILC2s. Data are shown as mean ± SEM. * *p* < 0.05, ** <0.01, *** *p* < 0.001 compared with PBS-treated or day 0 mice.

Previously we have reported the depletion of ILC2s in the gut of Sterne-infected mice [10]. To evaluate whether a similar depletion can be seen in the liver, we evaluated the number of ILC2s in the liver parenchyma (Figure S1). Surprisingly, instead of a depletion, we found a significant increase in the ILC2 population during the early stage of the disease; this increase was no longer apparent at day five post-infection (Figure 4A). Bacterial infections can be efficiently cleared by ILC3s, which secrete IL-17 and recruit neutrophils [31]; upon investigation, these cells did not significantly change in their percentage within the liver during infection (Figure 4B). The cytokines produced by ILC2s were also altered during the course of infection. IL-5 and IL-13 are two major cytokines produced by ILC2s to skew the immune response towards a Th2 phenotype. During Sterne infection, IL-5 expression was maintained at early stages of the disease; however, at day three post-infection, its expression declined, suggesting limited support to B cells for their survival and maintenance at Sterne-infected hepatic loci (Figure 4C). On the other hand, IL-13 expression declined very sharply in ILC2s; nonetheless, ILC2s recovered their IL-13 expression at day five post-infection (Figure 4D).

We previously reported a decline in the number of ILC2s [10] and their secretion of cytokines in the colons of mice infected with the Sterne strain of *B. anthracis*; however, here we report an increase in the ILC2 population within the liver. ILC2s express CXCR4, CXCR6, and CCR9 to reach peripheral sites [7,32]. The ligands for these receptors are CXCL12, CXCL16, and CCL25, respectively. We have evaluated transcription of these chemokines in the infected livers and spleens and found increased expression the genes (Figure 3), which suggests that these chemokines could allow for the recruitment of ILC2s to the liver to protect the damages resulting from Sterne infection.

## 3. Experimental Section

### 3.1. Mice and Ethics Statement

A/J mice were obtained from the Jackson Laboratory and bred in-house at the animal facility in the College of Veterinary Medicine at the University of Florida. Mice were provided food and water ad libitum. Mice were sacrificed at 6–8 weeks of age in accordance with the Animal Welfare Act and the Public Health Policy on Humane Care. All procedures were approved by the Institutional Animal Care and Use Committee (IACUC) at the University of Florida under protocol number 201107129 and all efforts were made to minimize animal suffering. Infected mice were monitored every 24 h and were humanely euthanized when signs of advanced infection (e.g., difficulty breathing) were noted.

### 3.2. *Bacillus anthracis* Spore Preparation and Mouse Infections

Spores were prepared from a toxigenic, non-encapsulated strain of *B. anthracis* (Sterne), as described previously [11], with the approval of the Institutional Biosafety Committee (IBC) at the University of Florida. To calculate final concentrations, serial dilutions (1:10) were grown in triplicate on brain-heart-infusion broth agar plates (Sterne) and colonies were subsequently counted. Mice were orally infected with Sterne spores (10^9^ spores/100 μL of PBS per mouse) after fasting for 4 h at the specific time points. For the survival study, mice (*n* = 13) were orally infected; infected mice were monitored, and deaths recorded during 14 days. For B cells studies, mice were orally infected; infected mice were monitored, and sacrificed at the corresponding days for B cells analyses in the liver and in the spleen. (*n* = 10 mice/group for PBS, day 1, 2 or 3 and *n* = 13 mice for day 5).

### 3.3. Quantitative Real-Time-PCR

Total genomic DNA was extracted from the hepatic and splenic tissues of the mice using gDNA MiniPrep kit (Zymoresearch, Irvine, CA, USA), following the manufactures’ instructions. qRT-PCR was performed on 100 ng of DNA template (SsoAdvanced SYBR^®^ Green Supermix, Bio-Rad, Laboratories, Inc., Hercules, CA, USA) to assess the dissemination of B. anthracis, Bifidobacterium, Bacteroidetes, and Enterobacteriaceae (PBS = 10 and day five, *n* = 13). Groups were normalized to the housekeeper Eubacteria group. A list of primers used and their sequences can be found in Table A1.

RNA was isolated from the liver and spleen using Aurum Total RNA mini Kit (Bio-Rad, Hercules, CA, USA), and cDNA was obtained via the iScript™ Select cDNA Synthesis Kit (Bio-Rad, Hercules, CA, USA). One µg of cDNA was used as a template for quantitative PCR via SYBR^®^ Green on a Bio-Rad CFX96 Real time system. mRNA levels are shown as the fold change increased over uninfected mice, and results were normalized to the housekeeper GAPDH gene. All primers that were used are found in Table A1 [9,10,33]. For statistical analyses, unpaired *t*-tests were performed using the relative expression levels of each gene at the specified time point compared to the relative expression level of the same gene in uninfected mice (PBS, days 1, 2, 3 post-infection: *n* = 10 mice/group and day 5 post-infection, *n* = 13 mice/group).

### 3.4. Isolation of Immune Cells for Flow Cytometry Analyses

Livers were perfused with 10 mL of phosphate-buffered saline (PBS) via the portal vein to remove circulating lymphocytes. Liver and spleen single-cell suspensions were prepared from whole tissue by mechanical disruption in RPMI-1640 with 5% fetal bovine serum (FBS). After washing, the subsequent suspension of the liver was disrupted through a 100-µm metal cell strainer and centrifuged through 40%–60% isotonic Percoll/RPMI 1640 gradient (GE-Healthcare, Uppsala, Sweden). For the spleen, erythrocytes were lysed with ACK lysis buffer and cells were washed twice using PBS with 1% FBS. Cells were counted and used for staining. Spleen and liver cell suspensions were stained using the LIVE/DEAD Fixable Aqua Dead Cell Stain Kit, then washed and incubated with an Fc blocking reagent (Miltenyi Biotec Inc., San Diego, CA, USA). A combination of different antibodies and their corresponding isotypes were used for labeled cell suspensions. Antibodies were purchased from eBiosciences (San Diego, CA), Biolegend (San Diego, CA, USA), and BD Pharmingen (San Diego, CA, USA): B220 (RA3-6B2), CD19 (6D5), IgM (RMM-1), IgD (11-26C), CD23 (B3B4), CD21/35 (7E9), CD45 (30-F11), CD11b (M1/70), CD5 (53-7.3), CD93 (AA4.1), CD90.2 (30-H12), IL33Ra (DIH9), GATA3 (L50-823), IL-7Rα (A7R34), CD25 (PC61), ICOS (7E.17G9), CD3 (145-2C11), CD11c (N418), RoRγt (B2D), IL6 (MP5-20F3), IL10 (JES5-16E3), IL13 (eBio13A) and IL5 (TRFK5). Lineage staining (Lin) used for the ILCs is composed of a combination of CD3 (17A2), CD11b (M1/70), CD11c (N418), Gr1 (RB6-8C5), CD19 (6D5) and B220 (RA3-6B2). Gating strategies are shown in the Figure S1. The Cytofix/Cytoperm kit (BD Biosciences, San Jose, CA, USA) was used for fixation and cellular permeabilization when intracellular staining was needed. Flow cytometric acquisition was performed using a BD LSRFortessa™ (BD Biosciences, San Jose, CA, USA). Data were analyzed with FlowJo software (Tree Star, Ashland, OR, USA).

### 3.5. Statistical Analyses

Data are represented as mean ± SEM. Statistical significance was calculated with Graphpad Prism software (LaJolla, CA, USA) using a two-tailed *t*-test for two group comparisons. Values of *p* < 0.05 were considered significant (* *p* < 0.05, ** *p* < 0.01, *** *p* < 0.001).

## 4. Conclusions

*B. anthracis* Sterne infection in A/J mice causes a polymicrobial infection due to the loss of intestinal barrier function [9]. Upon examination, data suggest that the polymicrobial infection within the liver might cause disruption of B cell and ILC2 functions. To fight the infection and to replace dysfunctional B cells and ILC2s, hepatic cells upregulate specific B cell and ILC2 chemokines. In the response to the chemokines, MZ-like B cells, B-1a cells, and ILC2s migrate into the hepatic parenchyma; however, they fail to clear the infection, potentially due to clevage of mitogen-activated protein kinases (MAPKs) [9,34,35] by the toxins released by the Sterne strain to suppress immune cell functions.

## Supplementary Materials

Supplementary materials can be accessed at: http://www.mdpi.com/2072-6651/7/9/3805/s1.

## Appendix

**Table A1:** List of primer sequences for Real-Time PCR analyses.

Gene/Organism	Sequence (5ʹ to 3ʹ)	References
*Bacteroides For*	GAGAGGAAGGTCCCCCAC	[36]
*Bacteroides Rev*	CGCTACTTGGCTGGTTCAG	
*Bifidobacterium For*	CGGGTGAGTAATGCGTGACC	[9]
*Bifidobacterium Rev*	TGATAGGACGCGACCCCA	
*Enterobacteriaceae For*	GTGCCAGCMGCCGCGGTAA	[9]
*Enterobacteriaceae Rev*	GCCTCAAGGGCACAACCTCCAAG	
*Bacillus anthracis For*	ATCACCAGAGGCAAGACACCC	[37]
*Bacillus anthracis Rev*	ACCAATATCAAAGAACGACGC	
*Cxcl13 For*	CAGAATGAGGCTCAGCACAGC	[9]
*Cxcl13 Rev*	CAGAATACCGTGGCCTGGAG	
*Cxcl11 For*	GGCGTCAAAACATGTGACATCC	[38]
*Cxcl11 Rev*	TGGCTGCATGTTCCAAGACAG	
*Cxcl10 For*	TCCCTCTCGCAAGGAC	[9]
*Cxcl10 Rev*	TTGGCTAAACGCTTTCAT	
*Cxcl12 For*	GAGCCAACGTCAAGCATCTG	[9]
*Cxcl12 Rev*	CGGGTCAATGCACACTTGTC	
*Cxcl16 For*	ACCCTTGTCTCTTGCGTTCTTCCT	[39]
*Cxcl16 Rev*	ATGTGATCCAAAGTACCCTGCGGT	
*Ccl25 For*	CCAAGGTGCCTTTGAAGACT	[33]
*Ccl25 Rev*	TCCTCCAGCTGGTGGTTACT	
*GAPDH For*	GGTGAAGGTCGGTGTGAACG	[9]
*GAPDH Rev*	CTCGCTCCTGGAAGATGGTG	
