# Supplementary material for: Impact of Gastrointestinal Bacillus anthracis Infection on Hepatic B Cells
Source: Toxins (Basel). 2015 Sep 22;7(9):3805–17. doi: 10.3390/toxins7093805 (PMC4591657; doi:10.3390/toxins7093805)
Supplement: Supplementary file 1 [file toxins-07-03805-s001.pdf]

Supplementary Information

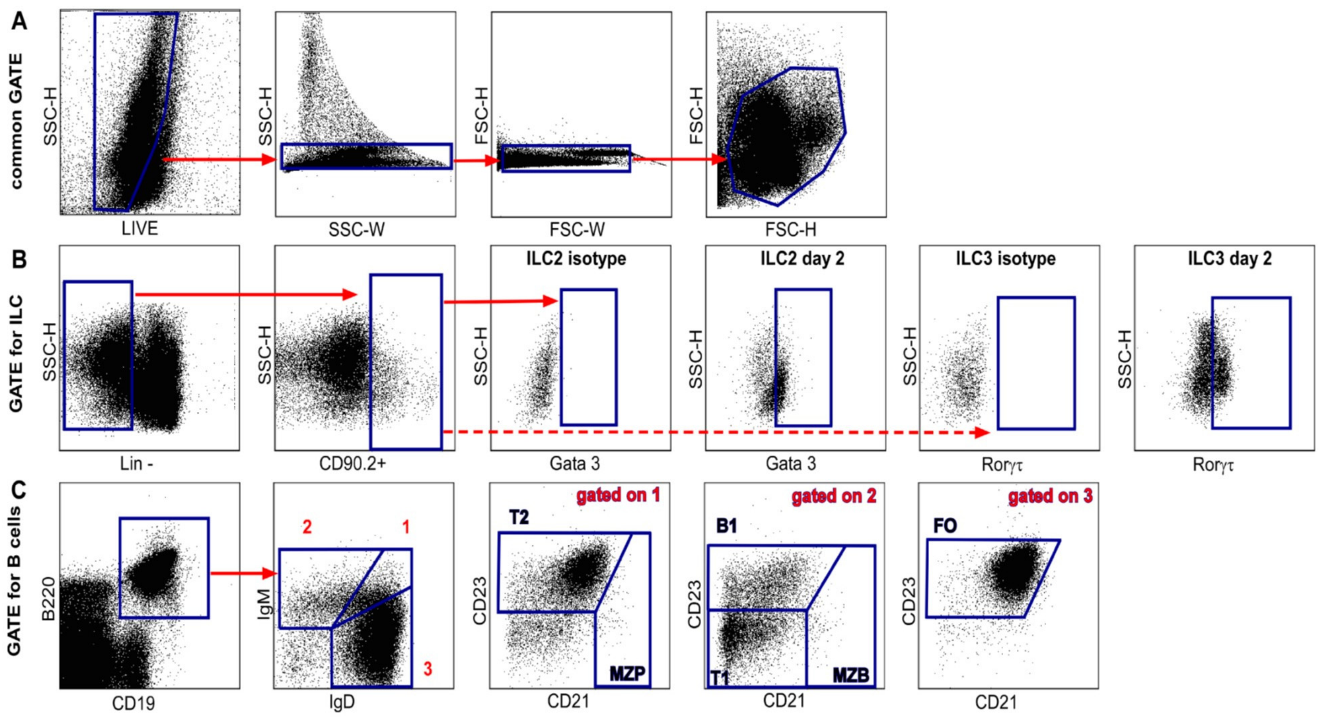

Figure S1. Gating strategies to define ILC2s and B cell subsets. A. Common gating strategy for the ILC and B cells subsets to depict doublets B. Gating strategy for ILC2s and ILC3s used in the study. C. Gating strategy to distinguish the B.

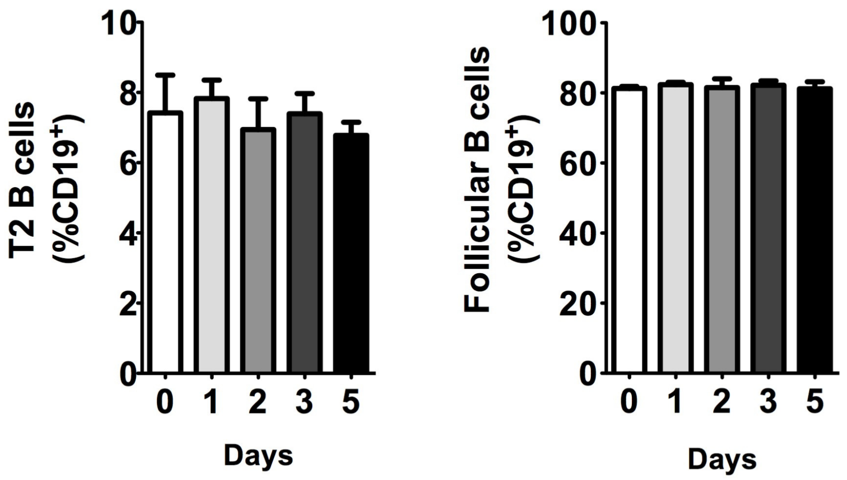

Figure S2. A/J mice were infected with 109 spores of B. anthracis Sterne, and liver leukocytes were isolated using Percoll gradients, as described in the Experimental Section. Isolated leukocytes were stained with different sets of antibodies, as depicted in Figure S1 to define Follicular and Transitional B cells. No statistically significant differences were found in these B cell subsets at the early phase of Sterne infection.
